# Studies on obesity-related atrial fibrillation in the past 20 years: A bibliometric analysis via CiteSpace and VOSviewer

**DOI:** 10.1097/MD.0000000000050429

**Published:** 2026-08-28

**Authors:** Bai Du, Jianming Yao, Yingshuo Niu, Chenlu Yuan, Yujie Luan, Xiaohan Zhang, Yuguo Song, Yuanhui Hu, Jiaran Li

**Affiliations:** a Department of Cardiovascular Diseases, Guang’anmen Hospital Jinan, China Academy of Chinese Medical Sciences, Jinan, Shandong, China; b Department of Cardiovascular Diseases, Guang’anmen Hospital, China Academy of Chinese Medical Sciences, Beijing, China; c Graduate School, Beijing University of Chinese Medicine, Beijing, China.

**Keywords:** atrial fibrillation, bibliometric analysis, CiteSpace, obesity, VOSviewer

## Abstract

**Background:**

Obesity is a well-established independent risk factor for atrial fibrillation (AF), and its association has been extensively investigated over the past 2 decades. This bibliometric analysis systematically evaluates research hotspots and emerging trends in the field of obesity-related AF.

**Methods:**

We extracted data from Web of Science Core Collection and conducted visualization analysis using CiteSpace (Drexel University, Chaomei Chen) and VOSviewer (Leiden University, Centre for Science and Technology Studies) software.

**Results:**

Among the 2621 included publications, annual publication counts demonstrated a consistent upward trend. The United States contributed the most studies, with the Mayo Clinic being the most prolific institution and Lip GYH being the most published author. Research in this field spanned multiple disciplines, with circulation as the most influential journal. The most frequent keywords were AF, obesity, and risk factors, along with 20 high-frequency and 25 burst keywords, indicating evolving trends.

**Conclusion:**

Through systematic bibliometric analysis, this study highlights key research hotspots and frontiers: cross-disease dimensional analysis, pathophysiological mechanisms, metabolic interventions, the obesity paradox, and AI applications in obesity-related AF. These findings provide a quantitative foundation for future research and offer valuable insights for investigators in this field.

## 1. Introduction

Atrial fibrillation (AF) is one of the most common arrhythmias encountered in clinical practice, characterized by disorganized electrical activity in the atria. Its prevalence increases with age, and it is estimated that approximately 50 million individuals worldwide were affected by AF in 2020.^[1]^ AF is associated with a reduced quality of life and serious complications, such as stroke and heart failure, posing a substantial burden on global healthcare systems. In recent years, the global rise in obesity has drawn increasing attention to the association between obesity and AF. A scientific statement from the American Heart Association (AHA) identifies excessive visceral adiposity as a major contributor to cardiovascular disease, with the risk of AF increasing by approximately 29% for every 5-unit increase in body mass index (BMI).^[2]^ Several studies have confirmed that the risk of AF is significantly higher in individuals with obesity compared to those without.^[3,4]^ The pathophysiological mechanisms underlying obesity-related AF are complex and involve metabolic disturbances, inflammation, and autonomic nervous system dysfunction.^[5–7]^

Despite extensive research on the association between obesity and AF, existing studies are fragmented and often concentrate on specific dimensions, such as pathophysiological mechanisms or therapeutic approaches. A comprehensive analysis of the broader research landscape remains lacking. Bibliometric analysis has become an essential tool for understanding research evolution and identifying emerging hotspots within a specific field. By quantitatively assessing variables such as publication volume, authors, institutions, and keywords, bibliometrics offers a structured perspective to better understand research development.^[8]^ Applying bibliometric analysis to the field of obesity-related AF enables a holistic assessment of the current research landscape, reveals evolving research directions and frontiers, and provides a scientific foundation for future investigation.

## 2. Methods

In this study, the Web of Science Core Collection was selected as the exclusive data source, as it is one of the most comprehensive and high-quality multidisciplinary citation databases, ensuring the representativeness and traceability of the literature. The data are secondary data, containing no identifiable personal information. Therefore, ethical approval and participant consent were not applicable to this study. The search strategy was defined as follows: TS = (Atrial Fibrillation OR Paroxysmal Atrial Fibrillation OR Familial Atrial Fibrillation OR Auricular Fibrillation OR Persistent Atrial Fibrillation) AND TS = (Obesity OR Adiposity OR Overweight) AND LA = (English). We imposed a language restriction on English-language publications. This restriction was motivated by 2 main considerations: firstly, English serves as the predominant language for international scholarly discourse and encompasses most high-quality, cutting-edge research; secondly, bibliometric analysis tools are not designed to perform equivalent keyword co-occurrence and cluster analyses in different languages, which could introduce significant methodological inconsistencies.

**Figure 1.**
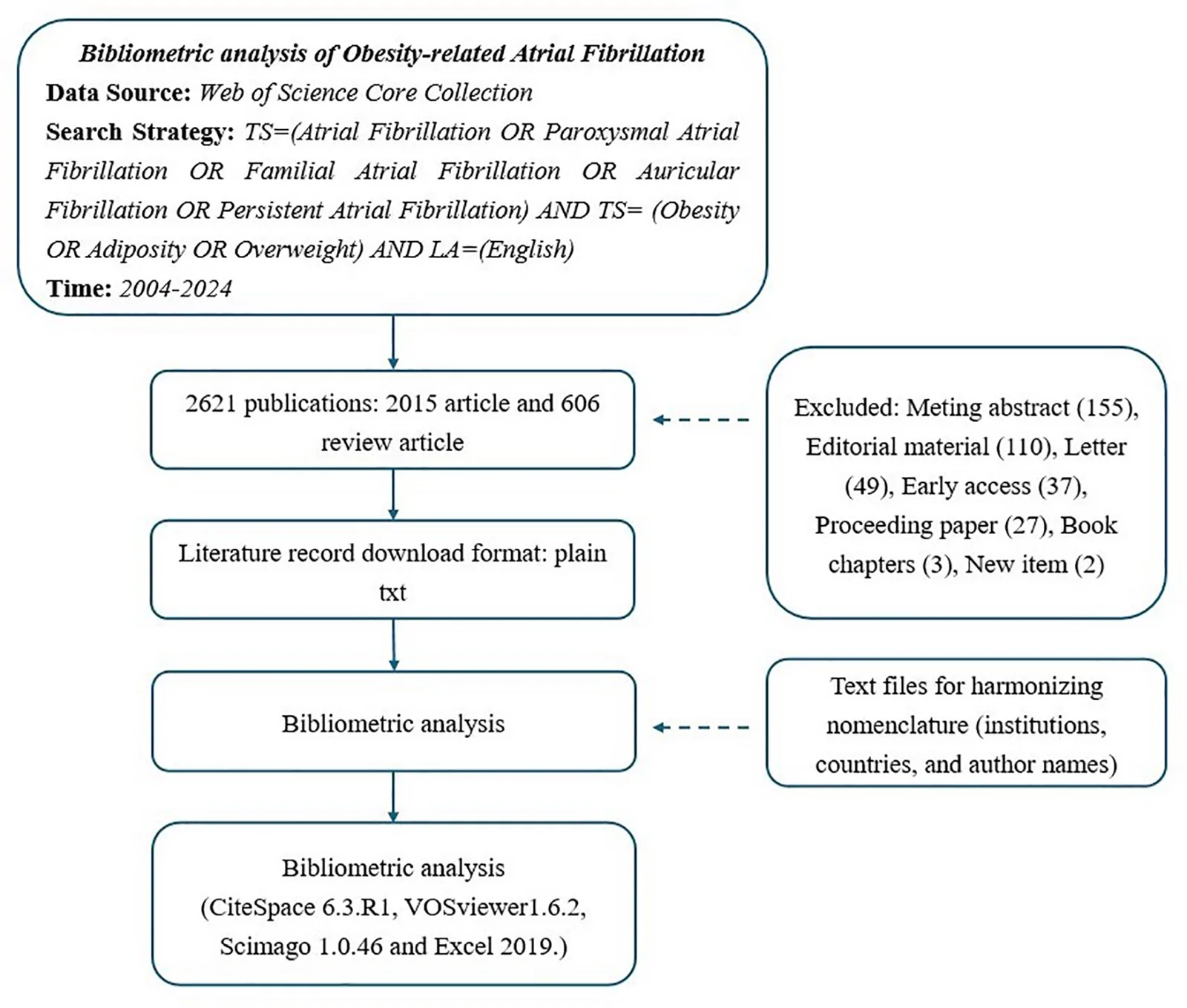
Flowchart of the analysis of obesity-related AF research. AF = atrial fibrillation.

A total of 2944 records were initially retrieved. After excluding meeting abstracts, editorial materials, letters, early access publications, proceedings papers, book chapters, news items, and similar types, 2621 records were included in the final analysis. The search process is illustrated in Figure 1. All records were exported in plain text format and labeled as “download_xxx” for subsequent analysis. Additionally, we implemented a rigorous standardization process prior to the bibliometric analysis. This involved creating text files to harmonize the nomenclature of institutions and countries. Similarly, author names underwent analogous standardization to ensure maximum data accuracy. The search was completed on October 18, 2024 to ensure data currency. The bibliometric and visualization analyses were conducted using CiteSpace, VOSviewer, Scimago Graphica, and Microsoft Excel 2019.

## 3. Results

### 3.1. Annual publication output

**Figure 2.**
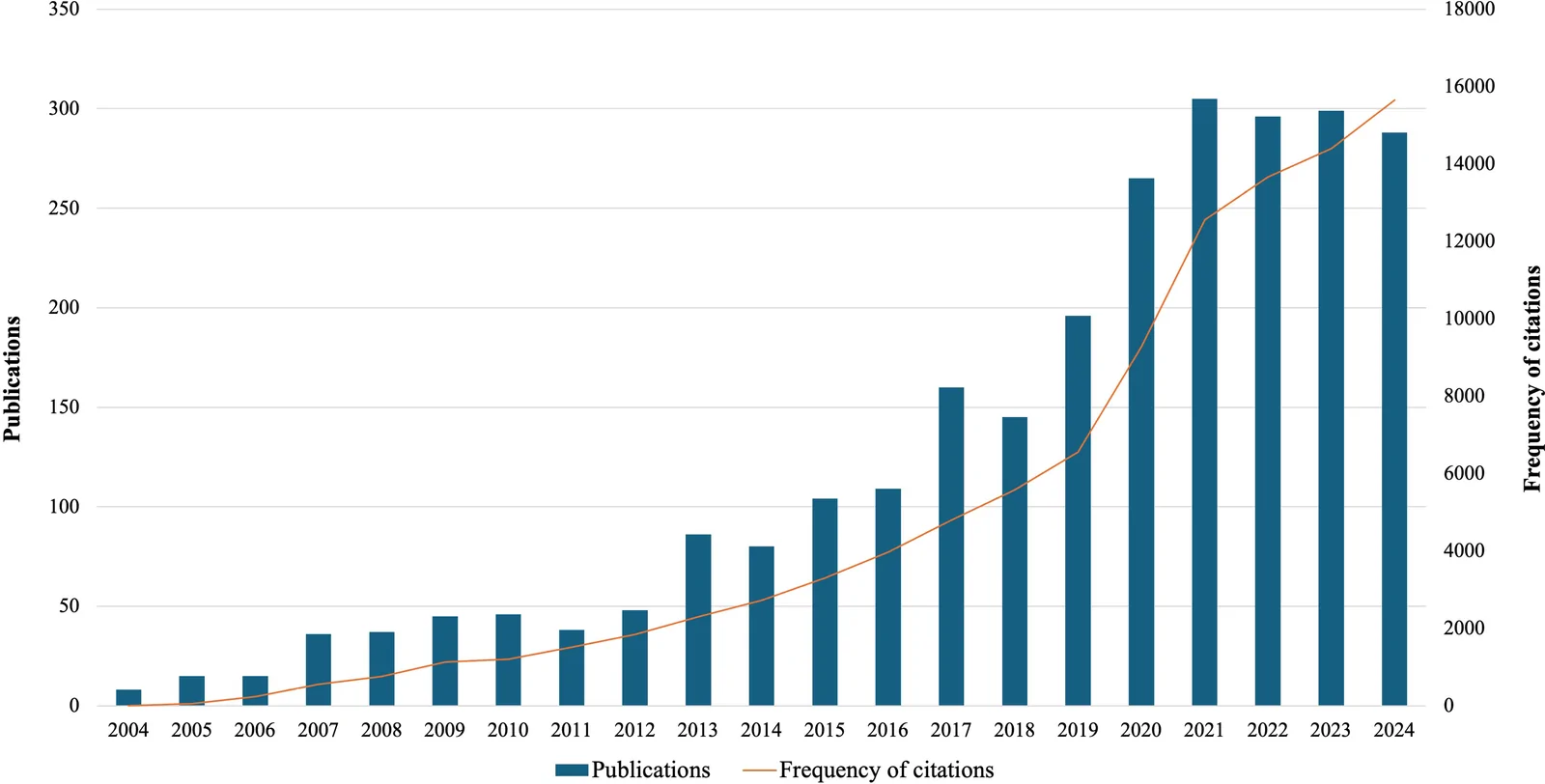
Number of annual publications and citation frequency in obesity-related AF research. AF = atrial fibrillation.

As shown in Figure 2, the volume of publications on obesity-related AF has demonstrated a steady upward trend over the past 2 decades. Since 2015, annual publication counts have consistently exceeded 100, with a peak of 305 articles reached in 2021. As of October 2024, a total of 2621 articles had been published. In parallel, citation frequency has also risen significantly, with >10,000 citations recorded since 2021. After excluding self-citations, a total of 65,341 citations were identified, yielding an average of 24.93 citations per article.

### 3.2. Countries and institutions

**Figure 3.**
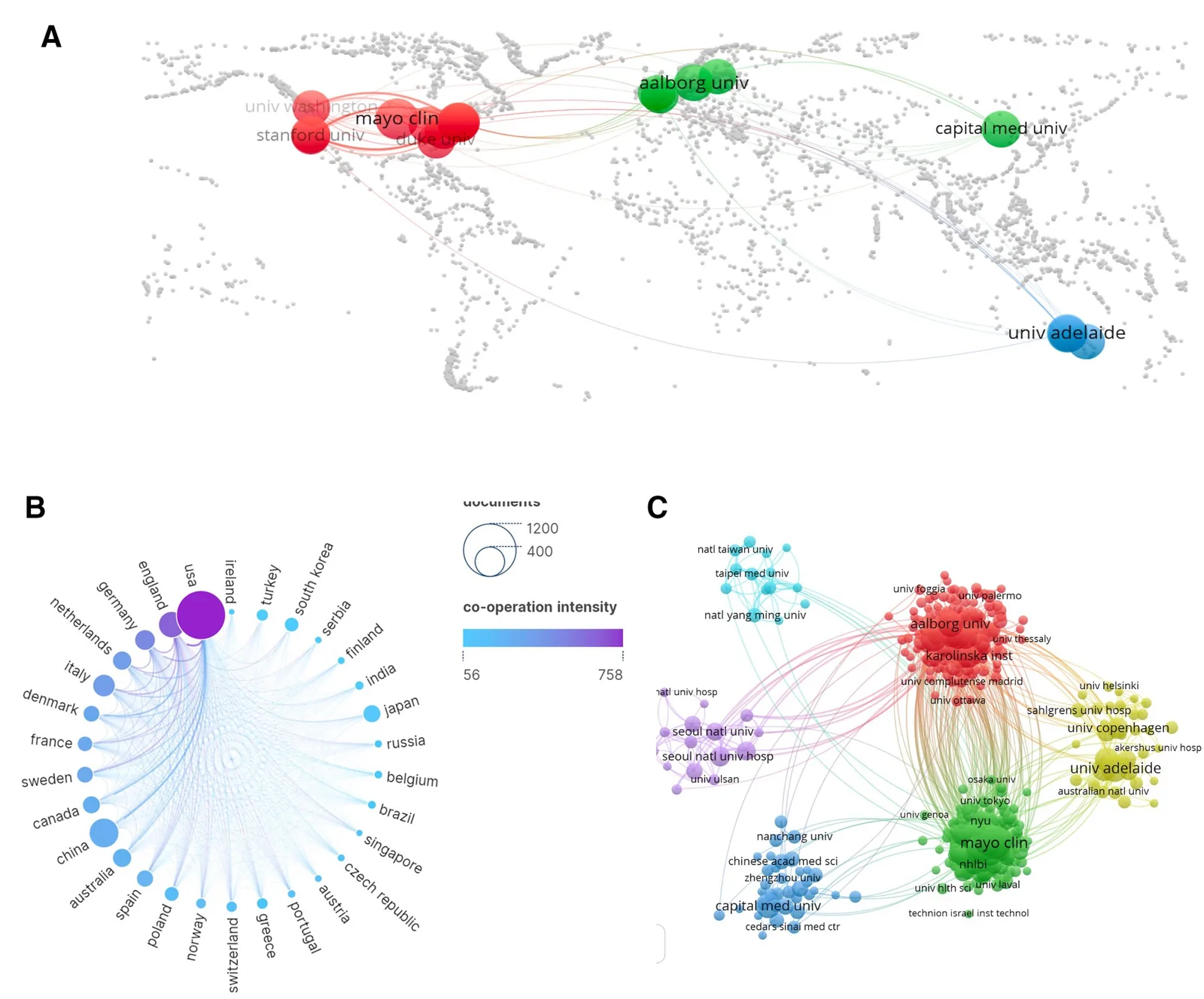
Network of countries and institutions engaged in obesity-related AF research. (A) Institutional collaboration map. (B) Country contribution map. (C) Institutional co-occurrence map. AF = atrial fibrillation.

To gain a deeper understanding of the contributions of various countries and institutions to obesity-related AF research, this study conducted a co-occurrence analysis of publication volumes from 109 countries and 4115 institutions (Fig. 3A and B). Table 1 lists the top 10 countries and institutions, ranked by the number of documents. The results show that the top 3 countries by number of records are the United States (977 articles), China (361 articles), and the United Kingdom (284 articles). In contrast, the top 3 institutions are the Mayo Clinic (75 articles), Harvard Medical School (63 articles), and Brigham and Women’s Hospital (59 articles), all based in the United States. Additionally, the United States exhibits the highest total citation count (55,361 citations), and only Italy achieves a centrality score >0.1. These findings suggest a notable “head effect” in this field, with most publications authored by scholars from a small number of countries and institutions, with relatively low levels of collaboration. Further analysis mapped institutions with more than 5 publications into a visualization network (Fig. 3C), revealing 6 collaborative clusters.

**Table 1:** The top10 countries and institutions in obesity-related AF research.

Rank	Country	Documents	Citations	Institution	Documents	Citations
1	United States	977	55,361	Mayo Clinic	75	348
2	China	361	7078	Harvard Medical School	63	563
3	United Kingdom	284	16,369	Brigham and Women’s Hospital	59	371
4	Italy	208	7983	Adelaide University	49	117
5	Germany	175	10,899	Massachusetts General Hospital	46	239
6	Netherlands	151	7153	Aalborg University	43	1257
7	Australia	150	12,683	Boston University	43	596
8	Canada	132	8258	Capital Medical University	42	785
9	Japan	132	3453	Cleveland Clinic	40	101
10	Spain	120	4182	Stanford University	39	3704

AF = atrial fibrillation.

### 3.3. Authors

**Figure 4.**
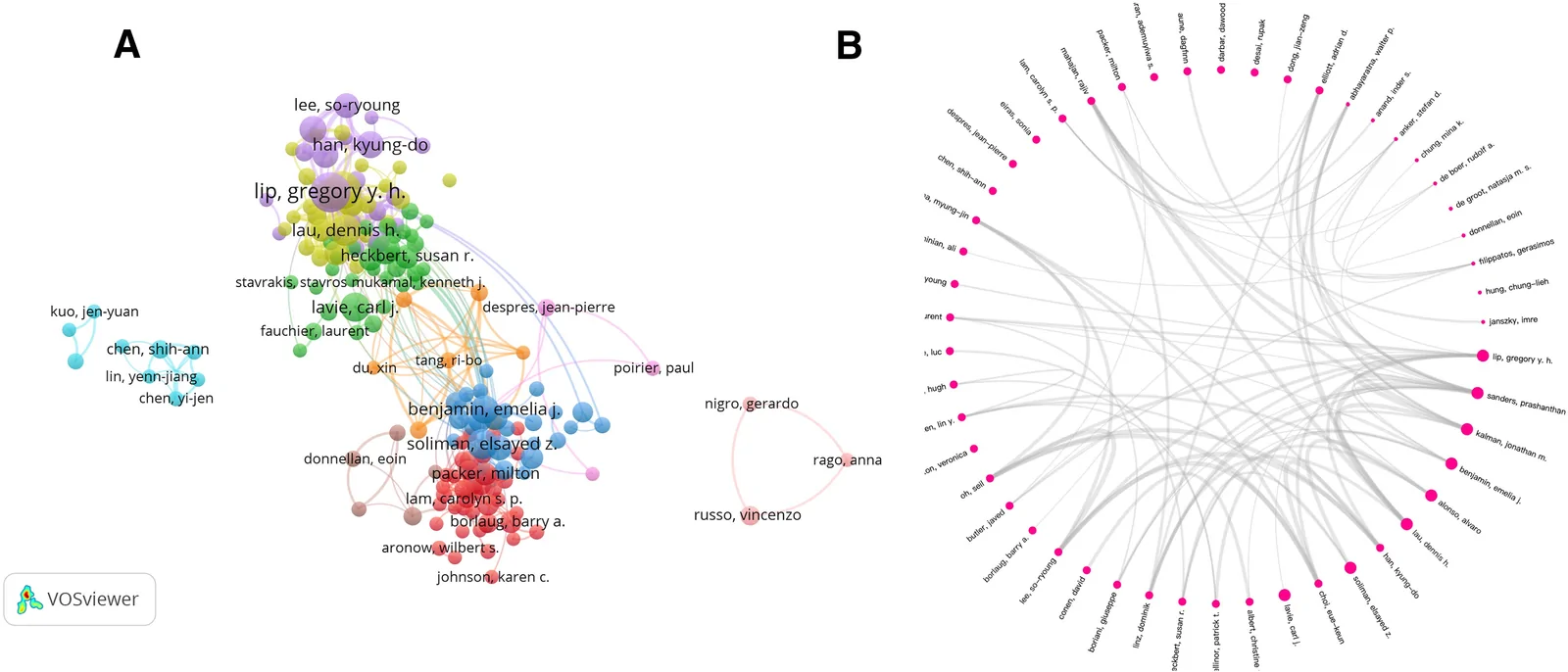
(A) Network of authors engaged in obesity-related AF research. (B) Top 50 authors’ co-occurrence. AF = atrial fibrillation.

As illustrated in Figure 4, the co-authorship network of authors with a minimum of 5 publications is presented. The top 5 authors by publication volume are Lip GYH (46 articles), Sanders P (41 articles), Kalman JM (27 articles), Lau DH (26 articles), and Oh S (19 articles). Among those demonstrating high productivity, Professor Lip GYH has the highest number of publications in this field, with a total of 506 citations. Meanwhile, Professor Kalman JM publications have garnered the highest total citations (3082), with an average of 114 citations per article. Professor Lip GYH has focused extensively on the impact of BMI on cardioversion outcomes and prognosis in AF patients,^[9,10]^ as well as the study of the obesity paradox in AF^[11]^; in contrast, Professor Kalman JM has conducted numerous randomized clinical trials on AF patients with obesity,^[12]^ and in recent years, has concentrated on the mechanisms linking persistent obesity and the development of AF.^[13]^

### 3.4. Co-cited journals

**Figure 5.**
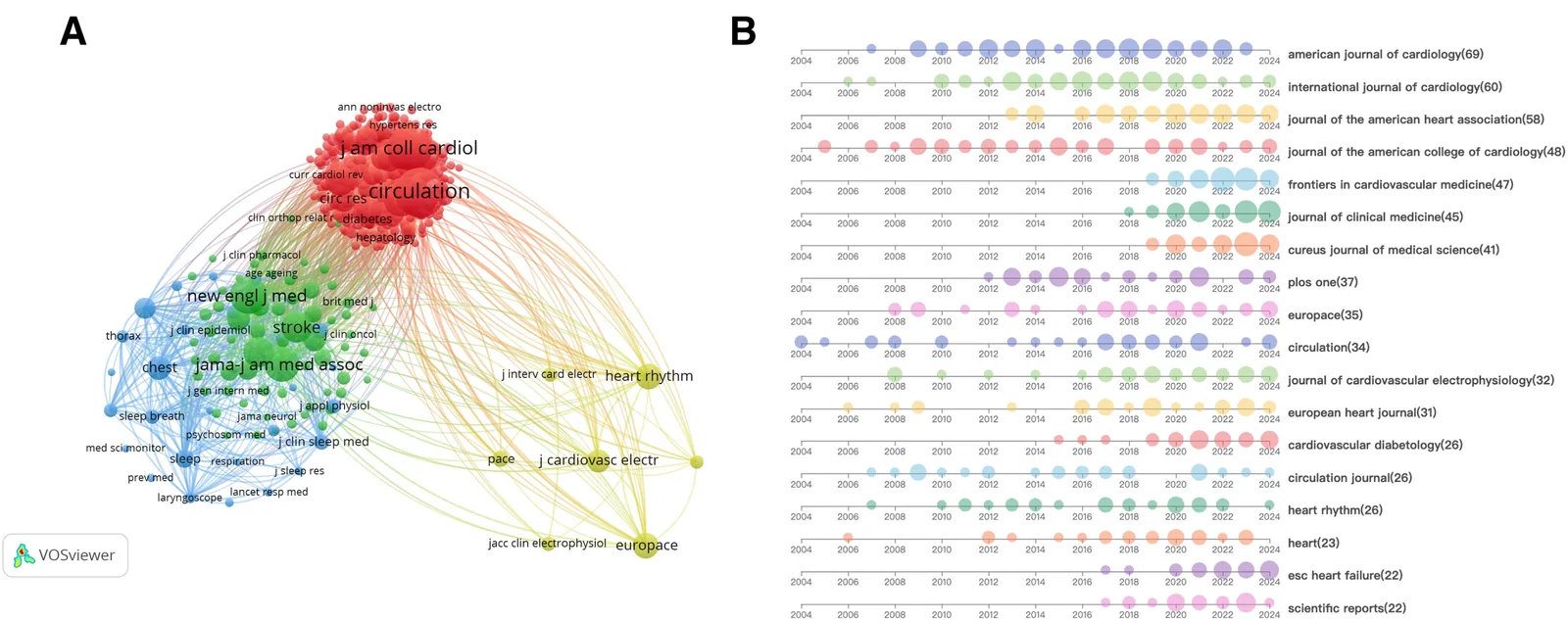
(A) Network of journals engaged in obesity-related AF research. (B) Timeline of core journals. AF = atrial fibrillation.

The 252 journals with a co-citation frequency >50 were visualized using VOSviewer (Fig. 5). The top 3 cited journals were *Circulation* (9417 citations), *Journal of the American College of Cardiology* (7319 citations), and *European Heart Journal* (4314 citations). All these journals are part of the red cluster, which focuses on the epidemiology, diagnosis, treatment, and analysis of risk factors for AF. The blue cluster focuses on metabolic diseases and endocrinology, with a particular emphasis on the pathophysiological mechanisms of obesity and the relationship between metabolic syndrome and cardiovascular disease. The green cluster, represented by journals such as *Stroke* and *Lancet Neurol*, focuses on the field of cerebrovascular disease, reflecting the analysis of the prognosis of obesity-associated AF research. Ultimately, the yellow cluster primarily comprises journals in the field of cardiac electrophysiology. The co-citation analysis of journals reflects the broadness of the research field.

### 3.5. Co-cited references

**Figure 6.**
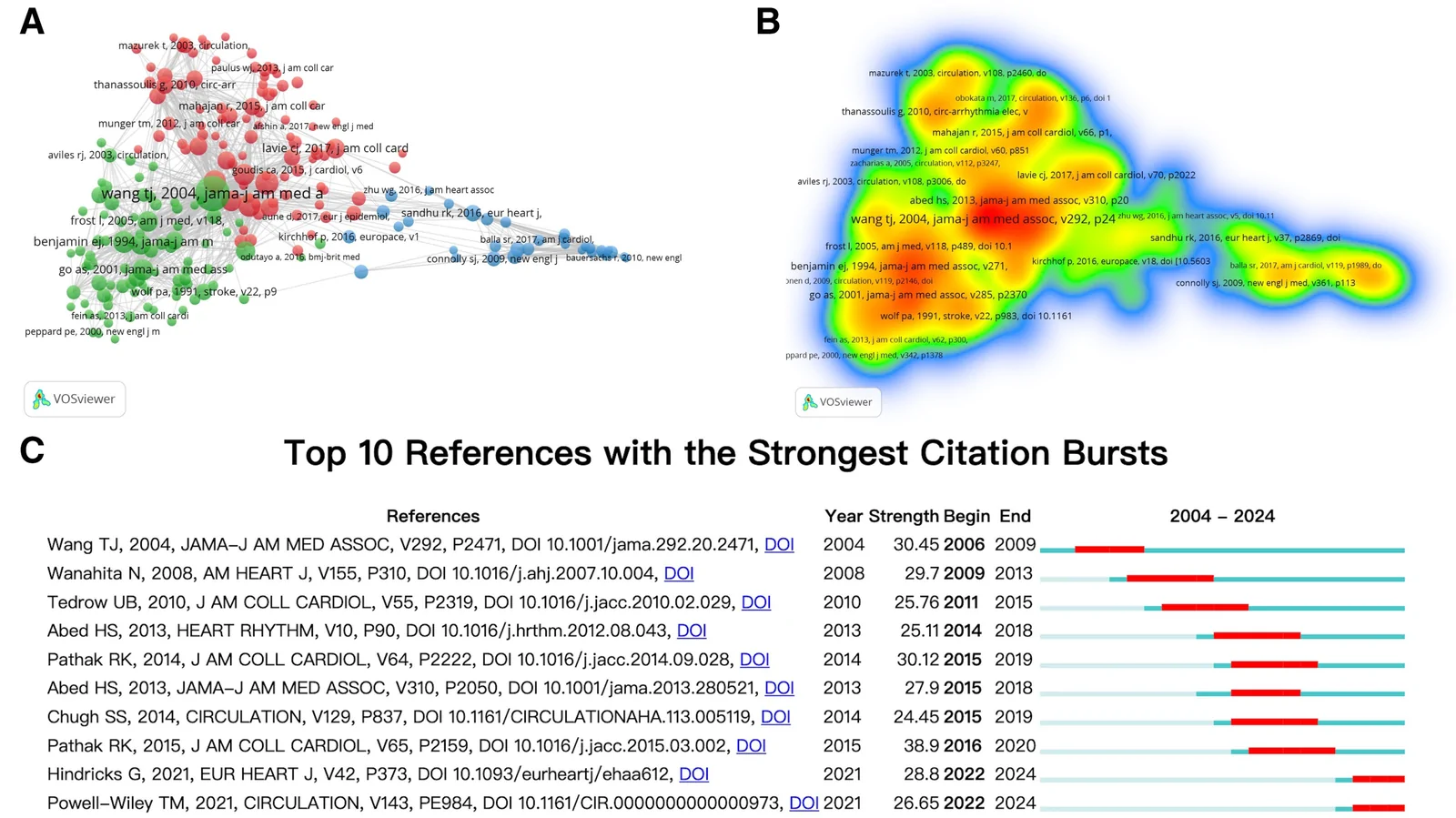
Network of co-cited references engaged in obesity-related AF research. (A) Occurrence map of references. (B) Density map of references. (C) The top 10 references with the strongest citation bursts. AF = atrial fibrillation.

Co-citation analysis identifies the relationship between 2 or more publications that are simultaneously cited by subsequent works. Using VOSviewer, a co-citation network analysis was conducted on 214 journals with >30 citations, with the co-citation network and density maps displayed in Figure 6A and B, respectively. As shown in Figure 6C, the most frequently cited reference is *Obesity and the Risk of New-Onset Atrial Fibrillation*^[14]^ (501 times). This landmark study, among the earliest in the field, established obesity as a critical risk factor for AF through a 13-year follow-up investigation. The study also identified left atrial enlargement as a potential mechanism underlying the association.

The reference with the highest citation burst intensity is *Long-Term Effect of Goal-Directed Weight Management in an Atrial Fibrillation Cohort: A Long-Term Follow-Up Study (LEGACY*)^[15]^ (227 times). This study offered a novel perspective on managing obesity-related AF by focusing on weight management strategies, thereby advancing the understanding of treatment approaches for this condition. In recent years, 2 co-cited references with sustained high citation bursts have emerged: the 2021 guidelines on the management of obesity-related cardiovascular diseases published by the European Society of Cardiology and the AHA.^[2,16]^ These guidelines, with burst intensities of 28.8 and 26.65, respectively, highlight the critical clinical implications and evolving importance of this research field.

### 3.6. Keywords

**Figure 7.**
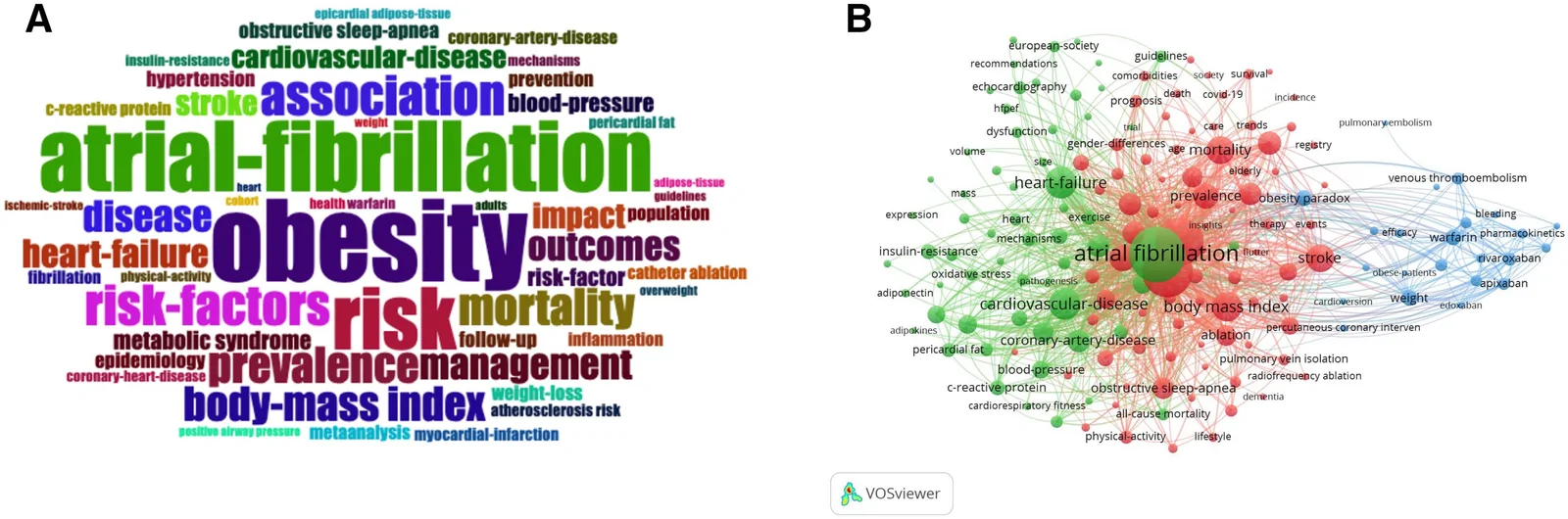
Network of keywords engaged in obesity-related AF research. (A) Word cloud of keywords. (B) Co-occurrence map of keywords. AF = atrial fibrillation.

**Figure 8.**
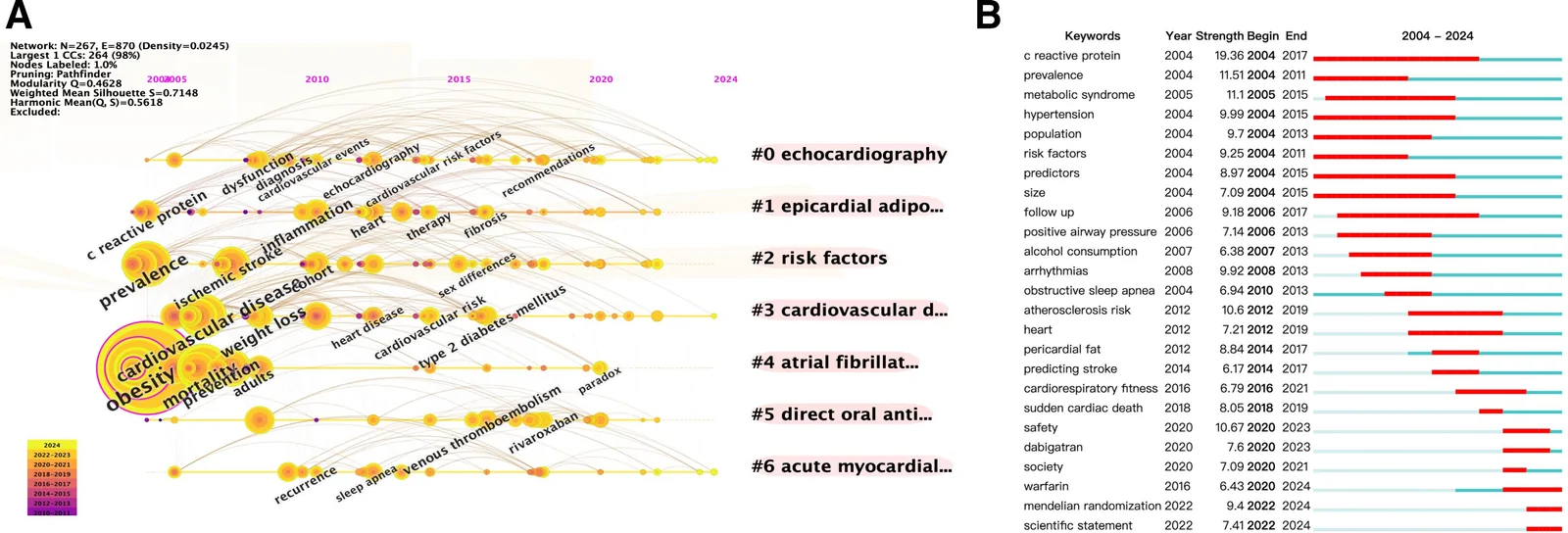
(A) Timeline map of keywords from 2004 to 2024. (B) Top 25 keywords with the strongest citation bursts.

As the core and a concise summary of an article, keywords play a crucial role in revealing the hotspots and trends within a research field. A co-occurrence analysis of 165 keywords with a frequency >20 was conducted, and a keyword cloud map was generated (Fig. 7A). The top 3 keywords are AF (1547 occurrences), obesity (1238 occurrences), and risk factors (1161 occurrences). Visualization of the keyword analysis is presented in Figure 7B, and the 20 most frequently mentioned keywords are detailed in Table 2. Then, we performed a cluster analysis of high-frequency keywords using Citespace, and a total of 7 clusters were obtained as shown in Figure 8A: #0 echocardiography; #1 risk factors; #2 epicardial adipose tissue; #3 cardiovascular disease; #4 atrial fibrillation; #5 direct oral anticoagulants; and #6 acute myocardial infarction.

**Table 2:** The top 20 keywords engaged in obesity-related AF research.

Rank	Keywords	Occurrences	Rank	Keywords	Occurrences
1	atrial fibrillation	1547	11	outcomes	274
2	obesity	1238	12	disease	262
3	risk factors	1161	13	management	261
4	heart failure	503	14	coronary-artery-disease	248
5	cardiovascular disease	498	15	epidemiology	245
6	body mass index	431	16	hypertension	238
7	stroke	400	17	obstructive sleep apnea	236
8	mortality	382	18	ablation	218
9	association	336	19	metabolic syndrome	217
10	prevalence	328	20	impact	214

AF = atrial fibrillation.

Burst keywords, defined as those exhibiting the highest frequency within a specific temporal framework, serve as critical indicators of research foci and thematic concentrations during a given period. Furthermore, the temporal dynamics of burst keyword intensity provide valuable insights for forecasting future research trends within the discipline. Through comprehensive extraction and analysis of specific keywords spanning the period from 2004 to 2024, we identified a total of 25 burst keywords (Fig. 8B). Notably, the keywords demonstrating the highest burst strength were C-reactive protein (19.36), prevalence (11.51), and metabolic syndrome (11.5), underscoring their pivotal role in advancing research on obesity-associated AF. Additionally, the recent emergence of keywords such as warfarin (6.43), Mendelian randomization (9.4), and scientific statement (7.41) over the past 3 years suggests a paradigm shift in research emphasis toward therapeutic interventions and clinical management strategies for obesity-associated AF.

## 4. Discussion

Unlike previous reviews that have predominantly concentrated on singular mechanistic aspects, our study employs knowledge mapping techniques to offer a quantitative and visualized view of obesity-related AF over the past 2 decades. For the 1st time, we systematically delineate 3 major research hotspots: cross-disease, pathophysiological mechanisms, and metabolic interventions. Burst keyword detection also helps forecast future hotspots, offering valuable direction for subsequent research.

### 4.1. The hotspots

#### 4.1.1. Cross-disease dimensional analysis

Obesity, a significant risk factor for AF, often manifests in conjunction with a range of metabolic and cardiovascular diseases, including type 2 diabetes mellitus, hypertension, and obstructive sleep apnea. These conditions form a complex pathological network. The results of the keyword co-occurrence analysis (Fig. 7) indicated that the most frequently occurring comorbid keywords were “hypertension,” “obstructive sleep apnea,” and “metabolic syndrome.” These keywords constituted a subset of studies centered on “metabolic co-morbidities – cardiac remodeling” (Fig. 7B). Among them, the term “metabolic syndrome” began to appear frequently around 2008, and then the research focus gradually shifted from single risk factors to multifactorial synergistic effects. The clustering network demonstrated that these comorbidities were associated with mechanisms such as “inflammation,” “epicardial adipose tissue,” and “insulin resistance,” among others. This finding suggests that the research focus has expanded from the epidemiological level to the molecular and organizational levels to explore the mechanisms.

Figure 8B illustrates the emergent word analysis conducted using CiteSpace, which reveals a notable surge in the emergence intensity of “safety” and “scientific statement” over the past 3 years. This observation supports increasing concerns about the connection between the cardiovascular and metabolic systems in the development of AF. For instance, a scientific statement from the AHA observes that obstructive sleep apnea, a condition intimately linked to obesity, significantly elevates the risk of AF onset and recurrence through mechanisms such as intermittent hypoxia, sympathetic activation, and atrial pressure load. In summary, the study of obesity-related AF research is becoming increasingly complex. Researchers are shifting their focus from studying individual risk factors to a broader examination of multisystem comorbidity, pathophysiological mechanisms, and comprehensive intervention strategies. This trend indicates that the prevention and management of AF should be approached from a multi-disease perspective. Furthermore, it establishes the foundation for developing a theoretical framework for “metabolic-AF syndrome.”

#### 4.1.2. Pathophysiological mechanisms

The evolution of mechanistic research in obesity-related AF illustrates a paradigmatic shift from epidemiological observation to biologically informed, target-driven investigation. This trend is seen not only in the research questions but also in the sophistication of research methodologies. Multiple Mendelian randomization studies^[17,18]^ have confirmed a significant causal link between obesity and AF, independent of conventional risk factors such as diabetes and hypertension. Early-stage studies primarily relied on conventional clinical data and regression models, limiting their ability to elucidate biological causality. In contrast, the recent adoption of high-throughput technologies – such as proteomics, metabolomics, single-cell sequencing, and multi-omics integration – has enabled a deeper understanding of the pathophysiological mechanisms underlying obesity-related AF.^[19]^

Notably, epicardial adipose tissue (EAT) has emerged as a key mechanistic hub in recent years.^[20]^ In obesity, EAT enlarges hypertrophically and is infiltrated by CD8+ T cells and M1 macrophages,^[19,21]^ leading to the excessive release of pro-inflammatory cytokines such as serum C-reactive protein, interleukin (IL)-6, IL-1β, IL-8, monocyte chemoattractant protein-1, and tumor necrosis factor-α. These factors contribute to changing the atrial microenvironment and stimulate fibroblasts, thereby promoting both electrical and structural remodeling of the atria. Furthermore, the clinical significance of inflammation is not limited to the initial pathogenesis of AF but extends to prognosis and recurrence. For instance, comparative studies on inflammatory markers, such as the C-reactive protein/albumin ratio or the uric acid/albumin ratio, have demonstrated a strong association with AF recurrence following cryoablation.^[22,23]^ Moreover, elevated uric acid/albumin ratio has been shown to independently predict new-onset AF in patients with ST-segment elevation myocardial infarction.^[24]^ These findings demonstrate that systemic inflammation, captured by inflammatory markers, serves as a critical mechanistic bridge linking obesity and AF onset.

Additionally, recent studies^[25,26]^ have confirmed significant alterations in gut microbiota composition in patients with obesity, characterized by reduced microbial diversity and increased pathogenic bacteria. This gut microbiota dysbiosis may impair intestinal barrier function and systemic inflammation through multiple mechanisms, thereby promoting AF onset.^[27]^ Microbial metabolites, such as trimethylamine oxide (TMAO), lipopolysaccharides (LPS), and short-chain fatty acids (SCFAs), are associated with systemic inflammation, oxidative stress, and immune activation, all of which may influence atrial substrate properties at a distance. Multiple studies have reported a significant association between TMAO and the risk of AF. For instance, Rienstra et al^[28]^ observed higher TMAO levels in patients with persistent AF compared to those with paroxysmal AF, suggesting a potential role in AF progression. However, the Prevención con Dieta Mediterránea study^[29]^ revealed no association between TMAO and AF, instead implicating TMAO precursors (e.g., choline).^[30]^ These discrepancies may stem from differences in study design, population characteristics, and methods for quantifying TMAO. LPS is significantly elevated in individuals with obesity versus healthy controls.^[25]^ Gut microbiota dysbiosis, particularly increased abundance of LPS-producing bacteria (e.g., Desulfovibrionaceae), plays a causal role in obesity-related AF pathogenesis.^[31]^ Impaired intestinal barrier function facilitates LPS translocation into circulation, causing metabolic endotoxemia that may indirectly promote AF through thrombotic mechanisms.^[29]^ LPS primarily activates the toll-like receptor 4/nuclear factor kappa-light-chain-enhancer of activated B cells signaling pathway and the NOD-like receptor family, pyrin domain-containing 3 inflammasome, increasing pro-inflammatory and profibrotic factors that drive systemic inflammation and oxidative stress.^[31,32]^ SCFAs, the primary metabolites of gut microbiota-mediated dietary fiber fermentation, consist of acetate, propionate, and butyrate.^[33]^ SCFAs suppress Ca^2+^/calmodulin-dependent protein kinases II and ryanodine receptor 2 phosphorylation,^[32]^ thereby preserving cardiac electrophysiological stability. Through these multifaceted mechanisms, SCFAs offer promising therapeutic potential for obesity-associated AF.

On the other hand, oxidative stress is also a key pathophysiological mechanism in obesity-related AF. It refers to the excessive accumulation of reactive oxygen species (ROS), which leads to cellular dysfunction. In obesity, adipose tissue expansion causes metabolic disturbances and ROS overproduction that exceed antioxidant defenses, initiating oxidative stress. This contributes to atrial remodeling via multiple pathways. ROS can enhance late Na^+^ current and early afterdepolarizations, increase L-type Ca^2+^ current and ryanodine receptor 2 activity, prolong action potential duration, and cause mitochondrial DNA damage and Ca^2+^ overload, disrupting cardiomyocyte electrophysiology and promoting electrical remodeling.^[34]^ Moreover, ROS activates fibroblasts and stimulates collagen deposition through the transforming growth factor-β pathway,^[35,36]^ upregulates inflammatory (IL-6 and IL-8) and profibrotic factors (matrix metalloproteinase 9, p38), and accelerates atrial fibrosis, contributing to structural remodeling and increasing AF risk.^[37]^ These insights are paving the way for the identification of novel therapeutic targets, such as blocking transforming growth factor-β signaling, modulating adipokine profiles, or restoring gut microbial balance.

In summary, research on the pathophysiological mechanisms linking obesity and AF is shifting toward a systems biology approach, characterized by integrating metabolic, inflammatory, and structural factors. This trend suggests that future studies should combine basic mechanisms with translational research to identify modifiable, mechanism-based targets for personalized prevention and treatment of AF.

#### 4.1.3. Metabolic interventions

As the mechanistic understanding of obesity-related AF continues to evolve, metabolic intervention has emerged as a promising therapeutic avenue, encompassing both pharmacological and non-pharmacological strategies.

Among pharmacological interventions, glucagon-like peptide-1 receptor agonists and sodium-glucose cotransporter 2 inhibitors – initially developed for treating type 2 diabetes mellitus – have garnered attention for their potential anti-arrhythmic effects. Evidence suggests that glucagon-like peptide-1 receptor agonists (e.g., liraglutide and semaglutide) may mitigate the risk of AF through multiple pathways, including the reduction of EAT, suppression of inflammation, and improvement of insulin sensitivity.^[38–40]^ Similarly, sodium-glucose cotransporter 2 inhibitors (e.g., dapagliflozin and empagliflozin) have been shown in experimental models to attenuate atrial oxidative stress, calcium overload, and mitochondrial dysfunction – mechanisms implicated in atrial electrical and structural remodeling.^[40]^ Additionally, gut microbiota modulation offers promising potential by enhancing metabolic and inflammatory profiles.^[25,32]^

Non-pharmacological metabolic interventions – such as exercise, low-carbohydrate or intermittent fasting regimens, and bariatric surgery – have also demonstrated favorable impacts on visceral fat distribution, autonomic tone, and adipokine profiles. For example, the Mediterranean diet (MD) is associated with a reduced cardiovascular risk. Studies show that MD lowers LPS levels and may modulate gut microbiota, thereby reducing atrial burden.^[41,42]^ The Prevención con Dieta Mediterránea trial^[43]^ confirmed that MD adherence significantly lowers AF risk, especially in individuals with obesity. Exercise is equally important. The LEGACY-AF study^[15]^ found that 150 minutes of moderate aerobic activity weekly reduced AF burden by 52%, and each 1 MET-h/wk increase lowered recurrence risk by 12%. The 2023 European Society of Cardiology guidelines recommend a combined approach of exercise, diet, and weight loss as 1st-line therapy for obesity-related AF (class I, level B).^[1]^ These interventions, which have been demonstrated to reduce visceral fat accumulation, improve the secretion profile of adipokines, and increase heart rate variability, are expected to become an essential component of primary or secondary prevention for obesity-related AF.

In summary, metabolic interventions have significant mechanism-targeted potential and application prospects in the field of obesity-related AF. Future research should concentrate on exploring the mechanisms underlying their effects on atrial electrical structure remodeling, while also examining the synergistic or alternative impact of different treatment modalities.

### 4.2. The frontiers

#### 4.2.1. Obesity paradox

The obesity paradox refers to the phenomenon that patients with obesity may exhibit better clinical outcomes in certain diseases. This phenomenon is particularly notable in cardiovascular disease and has recently gained widespread attention in the field of obesity-related AF.

The mechanisms underlying the obesity paradox remain unclear, and this phenomenon may be associated with multiple factors. Firstly, patients with obesity generally have more abundant metabolic reserves, with adipose tissue releasing free fatty acids and glycerol to provide energy under stress. Secondly, higher levels of protective adipokines (e.g., adiponectin) in individuals with obesity may improve outcomes through anti-inflammatory and anti-fibrotic effects.^[44]^ Additionally, patients with obesity enrolled in randomized controlled trials tend to be younger with fewer comorbidities, leading to better outcomes.^[45]^ Meanwhile, obesity-related AF patients are more likely to receive standardized anticoagulation treatments and rhythm control, potentially explaining their superior clinical outcomes.^[46]^

Clinical research offers complex yet illuminating evidence for the obesity paradox. Analyses of large randomized controlled trials, including the Apixaban for Reduction in Stroke and Other Thromboembolic Events in Atrial Fibrillation trial^[47]^ and the Atrial Fibrillation Follow-up Investigation of Rhythm Management study,^[48]^ have demonstrated that overweight or mildly obese AF patients (BMI 25–35 kg/m^2^) had a lower all-cause mortality rate and a significantly reduced risk of stroke and major bleeding versus the normal weight group. A meta-analysis^[11]^ by Gregory et al found that obesity-related AF patients on novel oral anticoagulants had superior efficacy and safety, with a 28% reduction in the risk of major bleeding compared to warfarin. Additionally, the obesity paradox is not unique to AF; it is a well-documented phenomenon in other cardiovascular conditions, most notably in acute coronary syndrome. A comprehensive meta-analysis examining the impact of the obesity paradox on mortality in acute coronary syndrome patients demonstrated that overweight and mildly obese patients had significantly lower all-cause mortality compared to their normal-weight patients, a finding that parallels observations in AF populations.^[49]^ This consistency across different cardiovascular pathologies suggests a shared, underlying protective mechanism.

However, this phenomenon was not fully replicated in observational studies and real-world data. Furthermore, meta-analyses indicated notable differences between randomized trials and observational studies, which may be related to study design, selection bias, and unmeasured confounders. It is noteworthy that the obesity paradox exhibits a discernible “threshold effect” and population-specific characteristics, which are not substantial in extreme obesity.

Future research should further investigate the mechanisms of the obesity paradox, integrating precision medicine approaches to develop novel strategies for managing obesity-related AF.

#### 4.2.2. Applications of artificial intelligence (AI) in obesity-related AF prediction

The rapid growth of AI and machine learning technologies has gradually expanded AI tools from traditional uses, such as automatic interpretation of electrocardiograms and image recognition, to more advanced research areas, including AF risk prediction, mechanism analysis, and personalized treatment planning.^[50,51]^ Especially in the field of obesity-related AF, AI provides innovative methods for integrating big data and predicting risks on an individual basis.

Firstly, AI can analyze extensive clinical cohorts that include multimodal data – such as demographic features, biochemical markers, metabolomics, and imaging data – to develop more refined models for predicting AF risk. Compared to traditional single-factor scoring systems, AI-based models can incorporate obesity-related features (e.g., BMI, visceral adiposity, and inflammatory marker profiles), resulting in more accurate and personalized risk assessments and enabling the early identification of high-risk groups for preventive measures.^[52]^ Secondly, in the field of electrocardiomics, AI algorithms can process raw 12-lead electrocardiogram signals or continuous data from wearable devices. This allows for the detection of “silent AF.”^[53,54]^ Additionally, when combined with atrial echocardiography,^[55,56]^ AI can provide a more precise assessment of atrial remodeling, aiding personalized decisions regarding catheter ablation or medication in patients with obesity. Finally, AI is expected to be used for dynamic risk monitoring. This involves combining wearable devices and mobile health platforms to continuously collect data, such as weight, heart rate, activity level, and sleep patterns. This will guide dynamic lifestyle interventions and adjustments to drug doses.^[57,58]^

Finally, the integration of multi-omics data (e.g., gut microbial metabolites and inflammatory cytokine panels) into AI pipelines is an emerging frontier. Early evidence suggests that incorporating these obesity-related systemic inflammation markers can further calibrate AF risk predictions, particularly in patients with discordant BMI and metabolic health phenotypes. Collectively, these developments position AI not as a generic predictive tool, but as a precision platform that bridges obesity-specific phenotypic heterogeneity to AF probability, paving the way for tailored surveillance and early lifestyle or pharmacological interventions. Future efforts should focus on strengthening the external validation of algorithms and exploring the integration of multi-omics data to enhance the predictive capabilities. This will further refine risk stratification and prevention strategies for AF in obese populations.

### 4.3. Limitations

This study has some limitations. First, the data source was limited to the WOSCC database, which has high academic authority but does not encompass all relevant literature, potentially resulting in the omission of some significant studies. Additionally, restricting inclusion to English publications may undervalue contributions from non-English speaking countries (e.g., China and Japan) in obesity-related AF research.

## 5. Conclusions

This study used bibliometric analysis to examine research trends in obesity-related AF. Using CiteSpace and VOSviewer, we analyzed publications from 2004 to 2024 across multiple aspects, including publication patterns, geographic distribution, leading authors, journals, references, and keywords. The findings highlight main research areas such as cross-disease dimensional analysis, pathophysiological mechanisms, metabolic interventions, the obesity paradox, and AI applications in obesity-related AF. These insights provide a quantitative base for understanding current priorities and guiding future research in the field.

## Author contributions

**Methodology:** Jianming Yao, Yingshuo Niu, Chenlu Yuan.

**Software:** Yujie Luan, Xiaohan Zhang, Yuguo Song.

**Writing – original draft:** Jiaran Li.

**Writing – review & editing:** Bai Du, Yuanhui Hu.
